# Supplementary material for: Natural statistics of human head orientation constrain models of vestibular processing
Source: Sci Rep. 2023 Apr 11;13:5882. doi: 10.1038/s41598-023-32794-z (PMC10090077; doi:10.1038/s41598-023-32794-z)
Supplement: Supplementary file 1 — Supplementary Information. [file 41598_2023_32794_MOESM1_ESM.pdf]

Natural statistics of human head orientation constrain models of vestibular processing

Supplementary material

Christian B. Sinnott[1,*], **Peter A. Hausamann**[2], **and Paul R. MacNeilage**[1]

[1]University of Nevada, Department of Psychology, Reno, 89557, United States of America
[2]Technical University of Munich, Department of Electrical and Computer Engineering, Munich, 80333, Germany
[*]csinnott@nevada.unr.edu

Bayesian model of perception of head orientation

Bayes' theorem states that the conditional probability of an event x given event y (posterior distribution) is equal to the product of the conditional probability of y given x (likelihood) and the probability of event x (prior), divided by the probability of event y (marginal).

$$P(x|y) = \frac{P(y|x)P(x)}{P(y)} \tag{1}$$

To model orientation perception, we substitute θ for x:

$$P(\theta|y) = \frac{P(y|\theta)P(\theta)}{P(y)} \tag{2}$$

$P(\theta)$ represents the prior probability across head orientation values based on the organism's prior experience. $P(y|\theta)$ is the likelihood, that is the probability of the current sensory information y given true head orientation θ. The posterior distribution $P(\theta|y)$ governs the perceptual estimate resulting from the model. It represents the conditional probability of true head orientation θ given current sensory information y. The marginal distribution in the denominator, $P(y)$, only serves to scale the product of the prior and likelihood distributions[1], so the equation can be simplified to:

$$P(\theta|y) \propto P(y|\theta)P(\theta) \tag{3}$$

In Eq. 3, the posterior distribution is proportional to the product of the likelihood and prior distributions. Our priors for head pitch and head roll models are kernel density estimates (KDEs) generated using our observed head pitch and head roll data. KDEs were generated using the `gaussian_kde` function in the `scipy.stats` library. Kernel bandwidth was determined using Scott's rule[2] (see Eq. 7).

In addition to these empirical priors, we also model the likelihood. Previous work shows increased variability of perceptual estimates as head orientation eccentricity increases, presumably due to vestibular sensory noise that increases with tilt angle. We model this noise on the likelihood in two ways.

$$f(\alpha,\sigma,\theta) = \alpha + \sigma \times |\theta| \tag{4}$$

The first is through a linear increase in noise as orientation eccentricity increases, shown in Eq. (4). Alpha is a constant representing a baseline level of noise determined from previous research[3,4], while sigma is a Weber fraction denoting the proportion of signal dependent noise. Alpha is added to the product of sigma and the absolute value of theta; the orientation angle. Second, we increase noise with eccentricity non-linearly by using a sinusoidal function and incorporating two constants, G and K, to better simulate the effect of utricular shear experienced during linear acceleration of the head[5] (Eq. (5)).

$$f(\alpha,\sigma,sin(\theta),K,G) = \alpha + \sigma \times K \times G \times sin(|\theta|) \tag{5}$$

Alpha, sigma, and theta are the same terms here as they are in the linear noise model. G represents the constant linear acceleration imparted by gravity, while K is a scaling constant determined in previous research[5]. We use both these equations to apply noise to the likelihood distribution used in each of our models.

To generate model predictions for each value of sigma and each tilt angle, we take the mean of the posterior distribution where the posterior is calculated as the product of the noisy likelihood function (with noise determined by σ and Eq. (4) and

(5)) and our empirical prior, according to Bayes' theorem (Eq. (3)). Both of our models only contain one free parameter which is the level of signal dependent noise denoted by σ. Both linear and utricular shear models are first fit to extant psychophysical data observing bias in roll perception[4] by selecting the sigma value that minimizes distance between the observed error from psychophysical data and predicted error from the model. We repeat the same process using pitch perception data[6]. Our metric for distance is residual square error (RSE), calculated as:

$$RSE = \sqrt{\frac{1}{n-2}\sum_{i=1}^{n}(y_i - \hat{y}_1)^2} \tag{6}$$

Modeling with smoothed empirical priors

To understand the effect of differing prior statistics on our model's predictions, we perform a subset of modeling with empirical KDEs that are generated using a Gaussian kernel with increasingly larger bandwidth h. In the current study, empirical priors used for modeling in the main text use a value of h that is calculated using Scott's Rule:

$$h = n^{(-1/(d+4))} \tag{7}$$

In this equation, n is equal to the length of the data to be smoothed, while d is equal to the number of dimensions in the data. Using this heuristic, h is approximately 0.04 for both empirical roll and pitch priors. By increasing the bandwidth used in KDEs of our roll and pitch priors, we can create versions of these distributions that have decreasing amounts of excess kurtosis and asymmetry relative to distributions generated with an h value determined by Scott's Rule (Fig. S1).

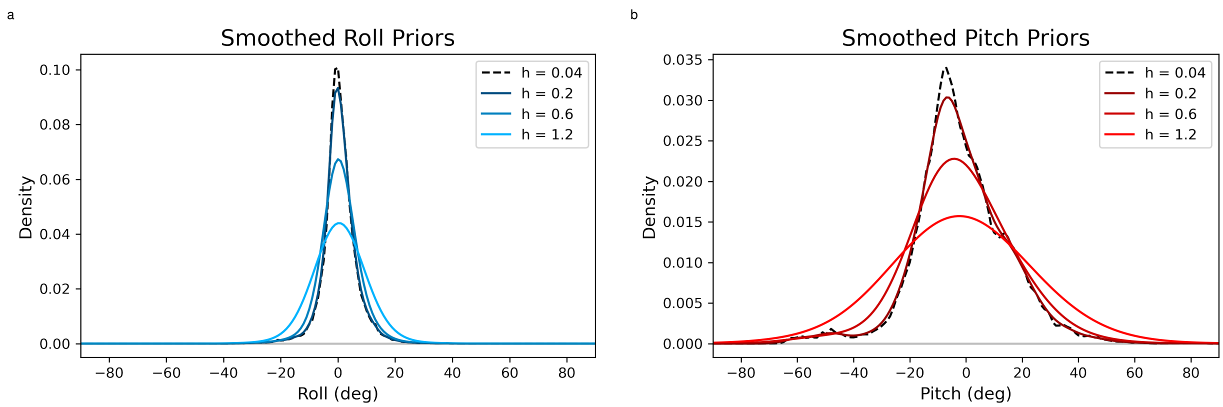

Supplemental Figure S1. Empirical roll (S1a) and pitch (S1b) priors smoothed with increasingly larger Gaussian kernel bandwidths (h). The dashed black line represents the KDE with bandwidth size calculated using Scott's Rule that was used in main analyses, while colored lines represent KDEs with increasingly larger bandwidths.

By using these smoothed distributions as priors in either of our models, we can probe model performance as a function of increased Gaussian kernel smoothing, and by proxy: reduced asymmetry and excess kurtosis. Like before, we find the value of σ that minimizes RSE between the model's prediction error using a given prior and observed psychophysical error from pre-existing work. Reducing excess kurtosis in this way for our roll distribution does not appear to drastically effect model fit, as every linear model using more smoothed versions of the empirical roll prior converged to use the same factor for multiplicative signal dependent noise (σ = 0.113) and had similar amounts of RSE (Fig. S2). Similar fits were achieved with all variants of prior using the utricular shear model (σ = 0.013-0.014).

An interesting, unanticipated consequence of this process of smoothing is that local extrema observed in posteriors using the original, least smoothed prior are also smoothed, resulting in a slight change to the underlying pattern of bias that our models predict. Given how likelihood distributions are generated, we conclude that this is driven by local extrema in the empirical prior. For roll in particular, a bimodal posterior is calculated at certain eccentricities which may explain the particular pattern of roll bias observed in our models (Fig. S3).

Performing the same smoothing and fitting process for pitch, we see a reduction in the qualitative asymmetry of the models' predictions. However, both variants of our static Bayesian observer model are not able to predict a pattern of bias coinciding with the observed pattern of pitch perception bias in previous work[6]. Regardless of the level of smoothing used on the empirical

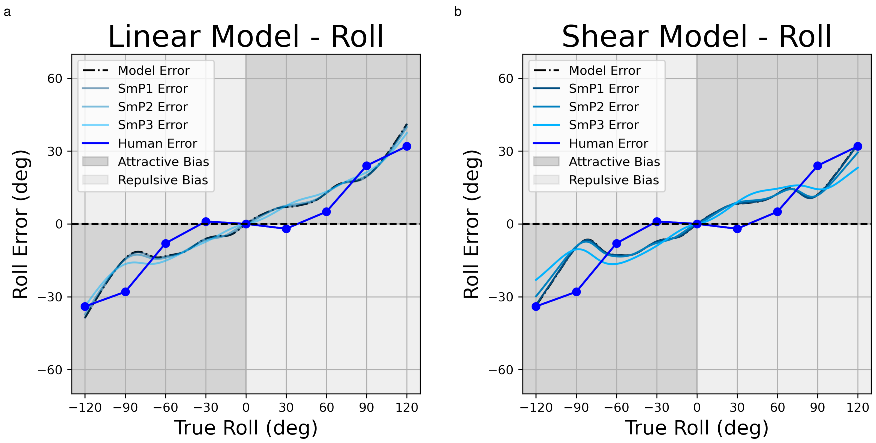

Supplemental Figure S2. Bayesian modeling results using roll priors of varying bandwidth for both linear (S2a) and shear (S2b) models. Original model results using the bandwidth determined by Scott's rule are plotted in dashed black; results using increasingly smoothed priors are plotted in light blue. Psychophysical data are plotted in dark blue.

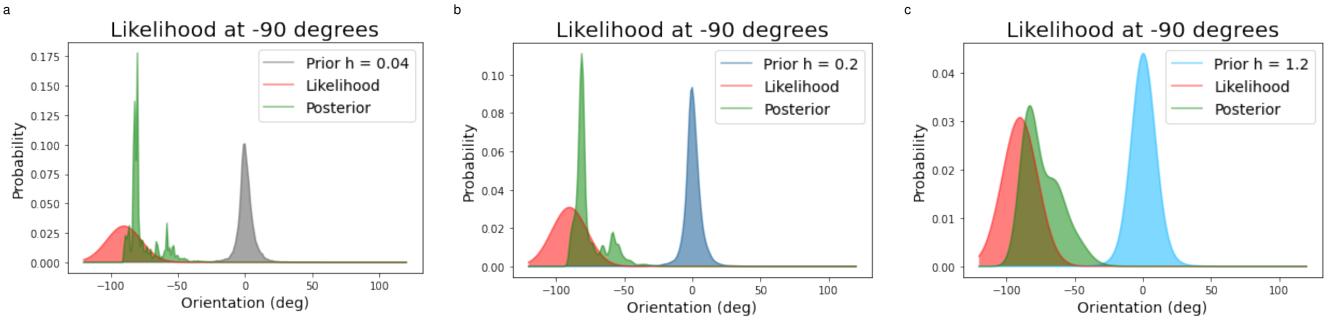

Supplemental Figure S3. Exemplar posteriors generated with likelihood at -90 degrees (left ear down) roll using priors generated using Gaussian kernels of varying bandwidth in the linear model. Signal dependent noise used in likelihood generation is the same across all three priors ($\sigma = 0.001$). Bandwidth increases from 0.04 at far left (S3a, same as prior used in main text), 0.2 in center (S3b), and 1.2 at far right (S3c).

pitch prior, our model minimizes to a set of predictions that have low error across nearly all pitch eccentricities. As with roll, every linear model using more smoothed versions of the empirical pitch prior converged to use the same factor for multiplicative signal dependent noise ($\sigma = 0.001$) and had similar amounts of RSE. Every shear model using various smoothed pitch priors demonstrated a similar pattern, both with respect to reduced asymmetry in the model prediction as well as a convergence to minimal signal dependent noise ($\sigma = 0.001$). All prior parameters, model parameters, and model performance metrics can be found in Table S2.

Gravitational and inertial acceleration power spectra during low versus high velocity epochs

After data were separated into separate high- and low-velocity epochs, we conducted power spectra analyses on both sets of epochs. Like before, crossing points were observed along all three axes, though the exact point of these crossing points differed from crossing points observed across all data. During low-velocity epochs, we observed crossing points at approximately 1.451 Hz (Fig. S5a), 0.775 Hz (Fig. S5b), and 0.761 Hz (Fig. S5c) along X-, Y-, and Z-axes, respectively. During high-velocity epochs, we observed crossing points at approximately 1.108 Hz (Fig. S6a), 0.553 Hz (Fig. S6b), and 0.53 Hz (Fig. S6c) along X-, Y-, and Z-axes. Additionally, power spectra estimated during high-velocity epochs showed a number of transient peaks through the frequency space along all axes, with a notable peak at approximately 2 Hz along X- and Z-axes corresponding with

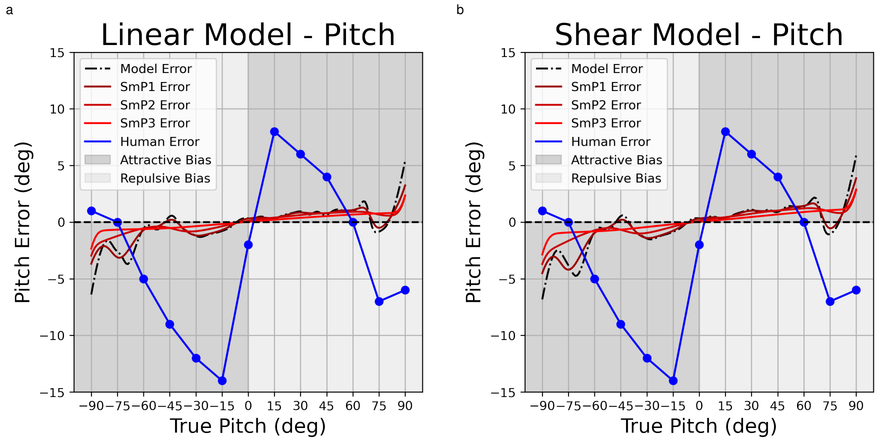

Supplemental Figure S4. Bayesian modeling results using pitch priors of varying bandwidth for both linear (S4a) and shear (S4b) models. Original model results using the bandwidth determined by Scott's rule are plotted in dashed black; results using increasingly smoothed priors are plotted in red. Psychophysical data are plotted in dark blue.

preferred stepping frequency[7]. No similar peaks were observed in the power spectra estimated during low-velocity epochs.

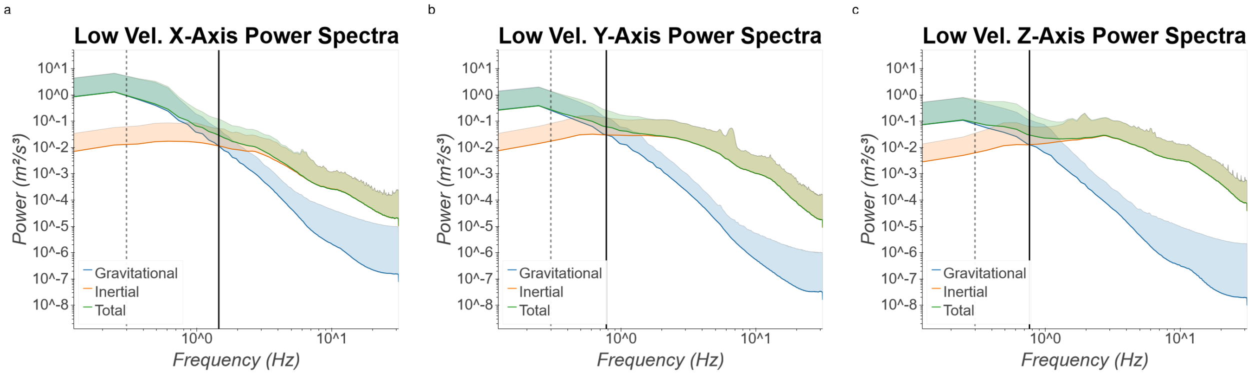

Supplemental Figure S5. Power spectra calculated from gravitational (blue), inertial (orange), and sum total linear acceleration (green) during low velocity (<0.75 m/s) epochs along X- (S5a), Y- (S5b), and Z-axes (S5c). Figure axes are log-scaled. Approximate crossing points in relative power between gravitational and inertial acceleration are observed at 1.451 Hz, 0.775 Hz, and 0.761 Hz for X-, Y-, and Z-axes, respectively.

Head orientation during low versus high velocity epochs

In addition to measuring head roll and head pitch across all participants and velocities, we measured head roll and head pitch across all participants during low- and high-velocity epochs. Given the proportion of total data that are comprised of low-velocity epochs (92.25% of all data), group-level distributions of head orientation as well as group-level KDEs of head roll and pitch look similar to their analogues across all data (Figure 2, main text). Conversely, the high-velocity head orientation distribution as well as high-velocity KDEs of head roll and pitch qualitatively differ from those measured across all velocity epochs (Fig. S8a). With respect to head roll, there appeared to be greater weight in the tails of the KDE relative to the KDE generated for roll across all velocities (Fig. S8b). Head pitch during high-velocity epochs appeared to be shifted further downward relative to head pitch across all velocities, both at a group and individual level (Fig. S8c). As high-velocity epochs primarily locomotion, this downwards shift is likely driven by concordant gaze-down behavior which functions to visually identify stable footholds[8,9].

We also calculated moments of pitch and roll distributions during low and high-velocity epochs across all participants. Descriptions of these moments as well as formulae used to calculate them can be found in Table S1. In general, these

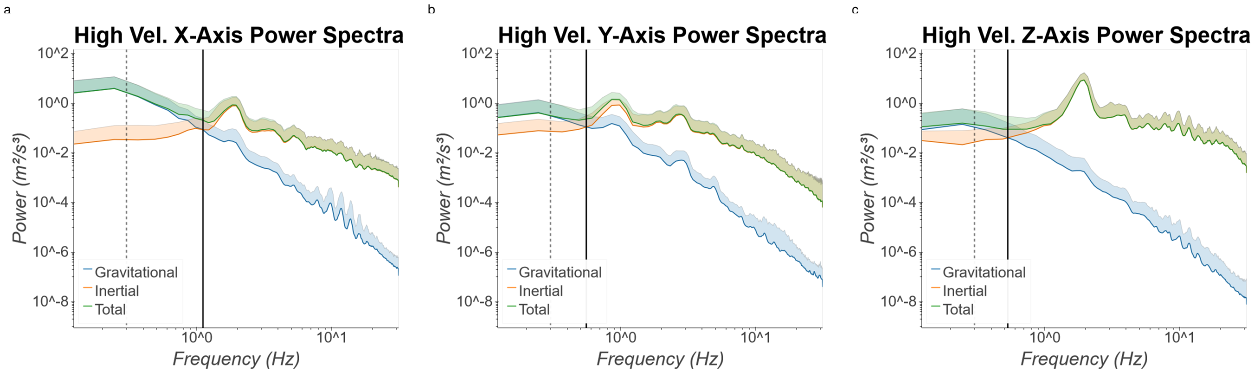

Supplemental Figure S6. Power spectra calculated from gravitational (blue), inertial (orange), and sum total linear acceleration (green) during high velocity (≥0.75 m/s) epochs along X- (S6a), Y- (S6b), and Z-axes (S6c). Figure axes are log-scaled. Approximate crossing points in relative power between gravitational and inertial acceleration are observed at 1.108 Hz, 0.553 Hz, and 0.53 Hz for X-, Y-, and Z-axes, respectively.

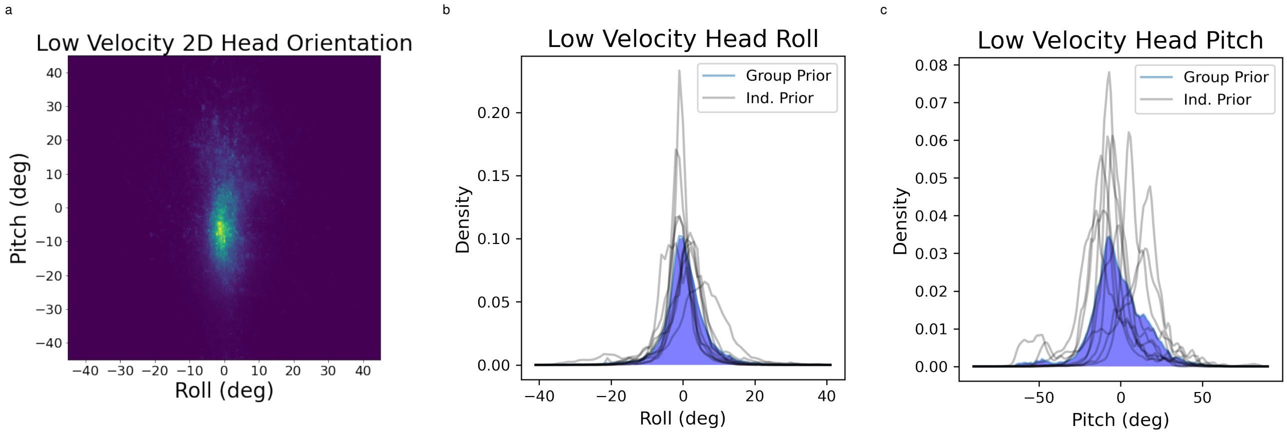

Supplemental Figure S7. 2D head orientation measured across all participants during low-velocity epochs. Marginal kernel density estimates (KDEs) for roll and pitch during low-velocity epochs are also plotted. The KDEs plotted in blue represent the distributions across all participants, while black traces represent KDEs for individual participants.

low-velocity histograms resembled histograms generated from both low- and high-velocity epochs. During low-velocity epochs, pitch was slightly biased downward (μ_1 = -1.574°) while average roll was closer to zero (μ_1 = 0.475°). Pitch had much higher variance (μ_2 = 288.39°) than roll (μ_2 = 39.673°). Both pitch (μ_3 = -0.036) and roll (μ_3 = 0.144) had little skewness during low-velocity epochs, while roll (μ_4 = 7.297) had greater excess kurtosis than pitch (μ_4 = 1.808).

Histograms generated from high-velocity data looked qualitatively different from histograms generated from both low- and high-velocity data, and this is reflected in moments calculated from high-velocity roll and pitch distributions. In particular, pitch appeared to show greater downward bias relative to the pitch KDE generated from all data. For high-velocity epochs, both roll (μ_1 = 1.832°) and pitch (μ_1 = -4.418°) were more biased away from upright. Pitch (μ_2 = 208.98°) continued to show higher variance than roll (μ_2 = 23.85°), and both pitch (μ_3 = 0.331) and roll (μ_3 = 0.02) showed low levels of skewness. Roll (μ_4 = 4.028) measured during these epochs continued to have higher excess kurtosis than pitch (μ_4 = 0.143).

We calculate moments of roll and pitch distributions for each subject as well (Tables S5, S6). Since we did not perform modeling using low- or high-velocity KDEs, we did not calculate their moments.

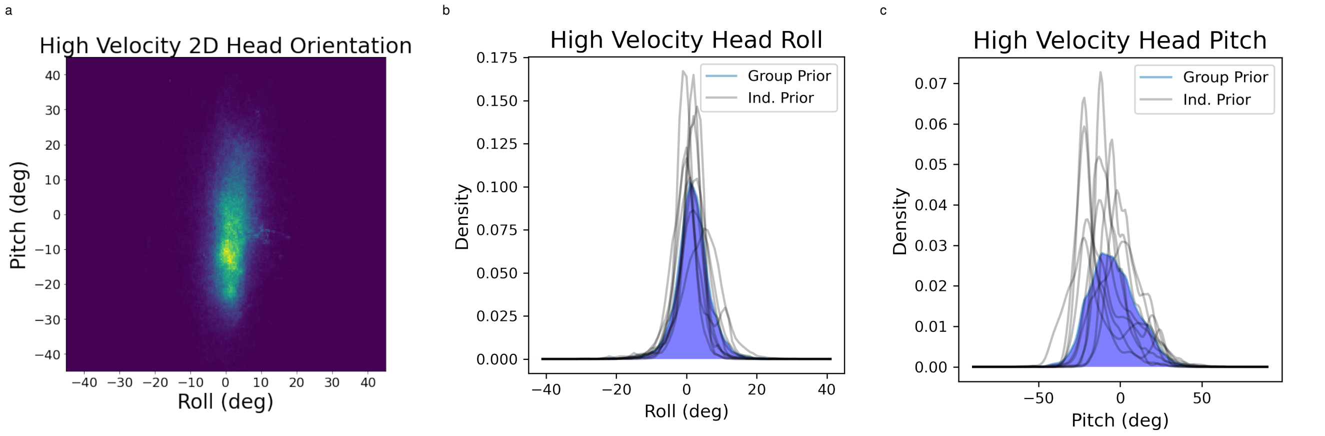

Supplemental Figure S8. 2D head orientation measured across all participants during high-velocity epochs. Marginal kernel density estimates (KDEs) for roll and pitch during high-velocity epochs are also plotted. The KDEs plotted in blue represent the distributions across all participants, while black traces represent KDEs for individual participants.

**Moment**	**Description**	**Formula**	**Low Vel. Roll**	**Low Vel. Pitch**	**High Vel. Roll**	**High Vel. Pitch**
Mean	The sum of a set of numbers divided by the count of that set.	$\mu_1 = \frac{1}{n}\sum_{i=1}^{n}$	0.475	-1.574	1.832	-4.148
Variance	The extent to which a set of numbers is spread out from the mean.	$\mu_2 = \frac{\sum_{i=1}^{n}(x_i - \bar{x})^2}{n-1}$	39.673	288.39	23.85	208.98
Skewness	The extent to which a given distribution is asymmetrical or otherwise lopsided.	$\mu_3 = \frac{m_3}{m_2^{3/2}}$ where $m_i = \frac{1}{N}\sum_{n=1}^{N}(x[n] - \bar{x})^i$	0.144	-0.036	0.02	0.331
Kurtosis	The weight of a given distribution's tails.	$\mu_4 = \frac{m_4}{m_2^{4/2}} - 3$ where $m_i = \frac{1}{N}\sum_{n=1}^{N}(x[n] - \bar{x})^i$	7.297	1.808	4.028	0.143

Supplemental Table S1. Descriptions, formulae, and values for first four central moments used to describe low-velocity and high-velocity pitch and roll histograms. Moments are calculated from group-level data plotted in 2d histograms in S7a and S8a
.

Prior Type	Bandwidth (h)	Model	Multiplicative Noise (σ)	RSE
Emp. Roll 1	0.04	Linear	0.113	6.438
Emp. Roll 2	0.2	Linear	0.113	6.418
Emp. Roll 3	0.6	Linear	0.113	6.38
Emp. Roll 4	1.2	Linear	0.113	6.261
Emp. Roll 1	0.04	Shear	0.013	8.346
Emp. Roll 2	0.2	Shear	0.013	8.392
Emp. Roll 3	0.6	Shear	0.013	8.74
Emp. Roll 4	1.2	Shear	0.014	9.612
Emp. Pitch 1	0.04	Linear	0.001	6.774
Emp. Pitch 2	0.2	Linear	0.001	6.687
Emp. Pitch 3	0.6	Linear	0.001	6.713
Emp. Pitch 4	1.2	Linear	0.001	6.835
Emp. Pitch 1	0.04	Shear	0.001	6.795
Emp. Pitch 2	0.2	Shear	0.001	6.712
Emp. Pitch 3	0.6	Shear	0.001	6.713
Emp. Pitch 4	1.2	Shear	0.001	6.845

Supplemental Table S2. Kernel density estimates parameters, model parameters, and goodness of fit metrics for model predictions depicted in S2a, S2b, S4a, and S4b.)

Prior Type	Location	Scale	Asymmetry	Model	Multi-plicative Noise (σ)	RSE
Gaussian Roll 1	0	5	N/A	Linear	0.001	5.736
Gaussian Roll 2	0	11.56	N/A	Linear	0.041	3.212
Gaussian Roll 3	0	15	N/A	Linear	0.062	3.016
Gaussian Roll 4	0	25	N/A	Linear	0.122	4.157
Gaussian Pitch 1	-7	12	N/A	Linear	0.001	7.105
Asymmetric Pitch 1	-18	22	2.5	Linear	0.001	6.782
Asymmetric Pitch 2	-19	32	5	Linear	0.001	7.199
Gaussian Roll 1	0	5	N/A	Shear	0.001	7.439
Gaussian Roll 2	0	11.56	N/A	Shear	0.007	4.585
Gaussian Roll 3	0	15	N/A	Shear	0.01	4.437
Gaussian Roll 4	0	25	N/A	Shear	0.021	4.157
Gaussian Pitch 1	-7	12	N/A	Shear	0.001	7.498
Asymmetric Pitch 1	-18	22	2.5	Shear	0.001	7.118
Asymmetric Pitch 2	-19	32	5	Shear	0.001	7.872

Supplemental Table S3. Distribution parameters, model parameters and goodness of fit metrics for simulation models using Gaussian and skew normal priors in main text. Location and scale parameters correspond with mean and variance of Gaussian distributions and other parameters were used to generate Gaussian and skew-normal distributions in Python 3 using Scipy library.

Subject	μ_{1Roll}	μ_{2Roll}	μ_{3Roll}	μ_{4Roll}	μ_{1Pitch}	μ_{2Pitch}	μ_{3Pitch}	μ_{4Pitch}
001	2.53	41.13	0.96	6.04	-5.8	200.74	0.7	1.05
002	0.43	34.46	-0.41	1.5	3.5	409.84	-0.85	0.19
003	0.73	30.57	-0.23	12.68	-11.11	462.81	-0.63	0.5
004	-0.48	11.8	-0.68	48.64	1.21	132.37	0.89	1.96
005	-1.81	27.41	-0.65	4.43	-2.05	158.67	1.41	4.01
006	5.45	59.04	0.08	4.9	10.77	177.56	1.54	4.79
007	-1.87	23.09	4.81	4.12	13.06	179.23	-0.05	1.1
008	-0.34	11.72	0.72	8.9	-5.69	75.3	1.38	4.84
009	-0.34	32.98	0.74	10.56	-8.21	168.42	1.7	4.71
010	-0.72	73.82	-1.65	3.46	-12.95	217.49	0.38	0.94

Supplemental Table S4. Moments of individual roll and pitch distributions across all epochs.

Subject	Low Vel. Data %	High Vel. Data %	μ_{1LVR}	μ_{2LVR}	μ_{3LVR}	μ_{4LVR}	μ_{1HVR}	μ_{2HVR}	μ_{3HVR}	μ_{4HVR}
001	80.7%	19.3%	2.56	45.85	0.98	5.49	2.41	21.25	0.32	6.93
002	98.6%	1.4%	0.41	34.67	-0.4	1.48	1.76	17.71	-0.83	3.36
003	94.8%	5.2%	0.7	31.55	-0.22	12.44	1.41	13.25	-0.69	9.33
004	92.9%	7.1%	-0.65	11.68	-0.66	52.89	1.76	7.98	-1.43	16.6
005	94.9%	5.1%	-1.88	27.89	-0.65	4.42	-0.55	17.02	-0.26	2.68
006	90.4%	9.6%	5.58	61.08	0.08	4.94	4.18	38	-0.2	1.83
007	92.2%	7.8%	-2.17	21.68	-0.13	4.71	1.34	26.82	0.35	1.56
008	97.8%	2.2%	-0.34	11.79	0.74	8.94	-0.55	8.67	-0.49	3.94
009	88.8%	11.2%	-0.43	34.37	0.84	10.78	0.36	21.01	-0.59	4
010	91.9%	8.1%	-0.96	77.19	-1.61	3.19	2.1	27.47	-0.77	2.29

Supplemental Table S5. Proportion of low- and high-velocity data for each subject, as well as moments of individual low- (μ_{nLVR}) and high-velocity (μ_{nHVR}) roll distributions.

Subject	Low Vel. Data Prop.	High Vel. Data Prop.	μ_{1LVP}	μ_{2LVP}	μ_{3LVP}	μ_{4LVP}	μ_{1HVP}	μ_{2HVP}	μ_{3HVP}	μ_{4HVP}
001	80.7%	19.3%	-6.98	202.95	0.89	1.61	-0.87	161.29	-0.02	-0.21
002	98.6%	1.4%	3.75	407.97	-0.88	0.27	-14.73	204.22	1.67	1.92
003	94.8%	5.2%	-10.82	477.27	-0.67	0.46	-17.09	127.05	1.63	4.21
004	92.9%	7.1%	1.81	129.99	0.91	2.1	-6.67	96.7	1.23	2.04
005	94.9%	5.1%	-1.76	151.86	1.55	4.58	-7.4	254.61	0.72	-0.51
006	90.4%	9.6%	11.44	177.55	1.62	5.02	4.43	133.16	0.88	1.21
007	92.2%	7.8%	14.04	170.27	-0.11	1.53	0.35	121.32	1.38	2.85
008	97.8%	2.2%	-5.66	75.45	1.38	4.89	-7.18	66.44	1.08	1.86
009	88.8%	11.2%	-8.6	162.13	1.85	5.64	-5.13	207.83	14.42	0.81
010	91.9%	8.1%	-12.94	203.23	-1.61	3.19	-12.97	381.13	0.59	-0.65

Supplemental Table S6. Proportion of low- and high-velocity data for each subject, as well as moments of individual low- (μ_{nLVP}) and high-velocity (μ_{nLVP}) pitch distributions.

References

1. MacNeilage, P. R., Ganesan, N. & Angelaki, D. E. Computational approaches to spatial orientation: From transfer functions to dynamic bayesian inference. *J. Neurophysiol.* **100**, 2891–2996 (2008).

2. Scott, D. W. *Multivariate Density Estimation: Theory, Practice and Visualization* (John Wiley & Sons, Inc., New York, New York, United States of America, 1992).

3. Vingerhoets, R. A. A., Medendorp, W. P. & van Gisbergen, J. A. M. Body-tilt and visual verticality perception during multiple cycles of roll rotation. *J. Neurophysiol.* **99**, 2265–2280 (2008).

4. de Vrijer, M., Medendorp, P. & Van Gisbergen, J. A. M. Accuracy-precision trade-off in visual orientation constancy. *J. Vis.* **9**, 1–15 (2009).

5. Schöne, H. On the role of gravity in human spatial orientation. *Aerosp. Medicine* **35**, 764–772 (1964).

6. Cohen, M. M. & Larson, C. A. Human spatial orientation in the pitch dimension. *Percept. Psychophys.* **16** (1974).

7. MacDougall, H. G. & Moore, S. T. Marching to the beat of the same drummer: The spontaneous tempo of human locomotion. *J. Appl. Physiol.* **99**, 1164–1173 (2005).

8. Matthis, J. S., Yates, J. L. & Hayhoe, M. M. Gaze and the control of foot placement when walking in natural terrain. *Curr. Biol.* **28**, 1224–1233.e5 (2018).

9. Pelz, J. B. & Rothkopf, C. Chapter 31 - oculomotor behavior in natural and man-made environments. In Van Gompel, R. P., Fischer, M. H., Murray, W. S. & Hill, R. L. (eds.) *Eye Movements*, 661–676 (Elsevier, Oxford, 2007).
